# Assessment of Image Quality in Digital Radiographs Submitted for Hip Dysplasia Screening

**DOI:** 10.3389/fvets.2019.00428

**Published:** 2019-12-03

**Authors:** Lilah Moorman, Helle Precht, Janni Jensen, Eiliv Svalastoga, Dorte H. Nielsen, Helle F. Proschowsky, Fintan J. McEvoy

**Affiliations:** ^1^Department of Veterinary Clinical Sciences, Faculty of Health and Medical Sciences, University of Copenhagen, Copenhagen, Denmark; ^2^Health Sciences Research Centre: Diagnosis and Treatment CONRAD, University College Lillebælt, Odense, Denmark; ^3^Research and Innovation Unit of Radiology, University of Southern Denmark, Odense, Denmark; ^4^Department of Radiology, Odense University Hospital, Odense, Denmark; ^5^Danish Kennel Club, Slorød Strand, Denmark

**Keywords:** visual grading analysis, digital radiography, PCA, image quality, hip dysplasia scheme

## Abstract

Digital radiography is widely seen to be forgiving of poor exposure technique and to provide consistent high quality diagnostic images. Optimal quality images are however not universal; sub-optimal images are encountered. Evaluators on hip dysplasia schemes encounter images from multiple practices produced on equipment from multiple manufacturers. For images submitted to the Danish Kennel Club for hip dysplasia screening, a range of quality is seen and the evaluators are of the impression that variations in image quality area associated with particular equipment. This study was undertaken to test the hypothesis that there is an association between image quality in digital radiography and the manufacturer of the detector equipment, and to demonstrate the applicability of visual grading analysis (VGA) for image quality evaluation in veterinary practice. Data from 16,360 digital images submitted to the Danish Kennel Club were used to generate the hypothesis that there is an association between detector manufacturer and image quality and to create groups for VGA. Image quality in a subset of 90 images randomly chosen from 6 manufacturers to represent high and low quality images, was characterized using VGA and the results used to test for an association between image quality and system manufacturer. The range of possible scores in the VGA was −2 to +2 (higher scores are better). The range of the VGA scores for the images in the low image quality group (*n* = 45) was −1.73 to +0.67, (median −1.2). Images in the high image quality group (*n* = 44) ranged from −1.52 to +0.53, (median −0.53). This difference was statistically significant (*p* < 0.001). The study shows an association between VGA scores of image quality and detector manufacturer. Possible causes may be that imaging hardware and/or software are not equal in terms of quality, that the level of support sought and given differs between systems, or a combination of the two. Clinicians purchasing equipment should be mindful that image quality can differ across systems. VGA is practical for veterinarians to compare image quality between systems or within a system over time.

## 1. Introduction

The major benefits of digital radiography are well accepted and include an increased tolerance of errors in exposure factor selection, avoidance of the negative environmental impact of film processing, ease of storage and retrieval, and if used correctly, improved image quality (1). For these reasons computed radiography or increasingly digital radiography dominate over film screen radiography in veterinary practice (2–4). This trend of digital radiography dominance is also seen in images submitted to the Danish Kennel Club for hip dysplasia screening. Over time, evaluators in this scheme have been under the impression that general image quality was related to the detector system manufacturer, which is known from the image metadata as the images are submitted in DICOM format. General quality is recorded by the screening evaluators for each image in the Danish Kennel Club database. This quality score is different to the hip evaluation score. The quality score is provided as a service to the veterinarians submitting images. It is based on a wide assessment including not only technical image quality issues, but also radiography technique such as patient positioning or beam centering. As such, this quality evaluation is not standardized and is not tested or further evaluated in this study. The evaluation was solely used to generate the hypothesis of a link between quality and detector manufacturer.

There is a perception that digital sensors and image processing and correct for all radiography errors, but in truth errors and artifacts also occur in digital systems (5). In the veterinary radiography literature there is lack of information on radiography quality control procedures specific to Digital radiography which are suitable for use in general practice. Quality control studies from the veterinary domain are limited in the literature. This sporadic occurrence of publications in this area suggests that there is poor awareness of the need to monitor image quality, both for reasons of diagnostic sensitivity and for maintaining radiation doses (to patients and, if present, technicians or owners) as low as reasonably practicable. A need for ongoing quality control in digital radiography is recognized in the human literature and a recent publication in the veterinary domain described work toward developing a quality control test specimen that may be pertinent to veterinarians (6). Procedures to achieve this may include rejected image analysis, exposure analysis, and artifact identification. All are suggested as being vital for the optimal operation of a department performing digital radiography (7). Reject image analysis in this context will include an assessment of image quality.

Digital image quality can be characterized by several parameters. Important among these are resolution, noise and artifact (8). Resolution describes the ability of the imaging system to separate features in the patient that are close to each other. These features may be close in the sense of physical space, in which case the term spatial resolution is used. This indicates the ability of the imaging system to display closely positioned features at separate locations. Alternatively two features may be close to each other in that they differ slightly in their ability to attenuate x-rays, in which case the term contrast resolution is used. It indicates the ability of the imaging system to display these similar but differently attenuating features at different points on a gray scale. An imaging system that can combine good spatial and contrast resolution will allow the radiologist to identify small objects that differ only slightly in their attenuating properties with surrounding tissue. Veterinary patients are often small relative to those encountered in human radiography and so may be particularly demanding of good spatial resolution. System noise may be seen as variations in image pixel value that are unrelated to the attenuation properties of the tissue being imaged. If an area that is expected to show uniform attenuation (e.g., muscle), shows random variations in pixel value (gray tone), this may be due to system noise. The inverse relationship between noise and the number of photons used to obtain the radiograph is important in radiography. The term “anatomical noise” refers to the role that normal anatomy may have in obscuring important pathology. Anatomical noise is considered to be the limiting factor in the detection of lesions in the thorax (9, 10). Artifacts can be thought of as features that are seen in an image and mask or mimic clinical features. Digital image artifacts can arise within the patient, or within acquisition hardware or software.

Methodologies for quantifying these parameters of image quality may be physical measurement, psychophysical evaluation or clinical assessment. Physical measurements include detective quantum efficiency (DQE) methods which are concerned with parameters such as modulation transfer function (MTF) and noise power spectrum (NPS). DQE methods are objective but are considered indirect methods of image quality. Descriptions of image quality from these physical perspectives give information about technical image quality, without any influence of human observers. Psychophysical methodologies of image quality assessment include the “contrast detail” analysis. Observers are asked to score images from phantom objects and the results provide quantitative assessments of low contrast and small detail measurements. These measurements correlate well with performance measurements in chest radiography (11). Both the physical and psychophysical methodologies however are based on measurements from phantom objects and can be criticized for not reflecting realistic clinical image environments (12). Performing and interpreting objective physical measurements of image quality are likely beyond the veterinary practitioner who may be considering the purchase of new imaging equipment, or concerned with maintaining and improving image quality over time with existing equipment as part of a quality control procedure. Visual grading analysis (VGA) is a clinical assessment, and is accessible to the veterinary practitioner for image quality audits. It is based on the ability of observers to detect and perceive predefined image criteria (13). It is an image evaluation methodology that is reported to be have attractive simplicity and powerful discriminating properties (14, 15). A VGA may be performed using absolute or relative grading. In the former, assessors score the degree to which specific image criteria are met. Relative grading on the other hand compares specific image criteria in the image being assessed to the same criteria on a reference image. This latter form of VGA was used in this study and our 5 point grading scale is typical. In relative VGA, a high score simply indicates the degree to which the image examined is better than the reference. Both the reference and test images may be excellent or both may be poor.

The aims of this study are to confirm the suspected association between quality grades in the kennel club records of hip dysplasia screening radiographs and the system manufacturer, and to rank manufacturers according to image quality. If the suspected association is confirmed, this ranking will be used to create two groups of manufacturer by image quality. Visual grading analysis will then be used to test the hypothesis that images chosen at random from each of the two image manufacturer groups differ with respect to image quality assessed by VGA. The null hypothesis being that there will be no difference in VGA quality scores between the two groups.

## 2. Materials and Methods

Digital radiography images (16,360) submitted during the period 2012 to 2017, to the Danish Kennel Club for hip dysplasia screening were retrieved from a patient archiving and communication system (PACS). For each image the manufacturer name (available as metadata in the header of each image) was retrieved, as was a kennel club quality grade awarded at the time of hip dysplasia grading. The grade uses an ordinal scale with three categories 1, 2, and 3 and was stored in the kennel club database. A kennel club quality grade of “1” is applied to images of satisfactory quality and grades of “2” and “3” applied to images with increasing degrees of technical faults, but are nonetheless of diagnostic quality for the purposes of awarding a hip dysplasia screening score. Technical faults in this context include suboptimal image contrast and spatial resolution, the presence of noise and also artifacts unrelated to the detector system such as labeling and positioning errors. The images were ranked according to their kennel club image quality grade. Statistical analysis was performed on these data as part of the hypothesis generating process to confirm that an association exists in the database between manufacturer and quality grade and also to determine mean kennel club quality grade for each manufacturer. The quality grade was then used to create a list of manufacturers ranked by image quality. Images from the top three ranked manufacturers were assigned to a group (high quality), and images from the bottom three manufacturers were assigned to a group (low quality), for VGA analysis as described below. This study was carried out in accordance with the commitments contained in the Basel Declaration and adhered to the General Data Protection regulations of the European Union. The protocol was approved by the local Ethics and Administration Committee, Department of Veterinary Clinical Sciences, University of Copenhagen.

### 2.1. Visual Grading Analysis

The three manufacturers with the three highest average kennel club quality grades (high quality group) and the three with the three lowest grades (low quality group) were selected for the VGA. Fifteen images from each manufacturer were randomly selected for evaluation, resulting in a total of 45 images per group (total 90 images). The null hypothesis was that there would be no difference in VGA scores between groups. In addition three images from each manufacturer (i.e., 18 images) were duplicated. These duplicates were combined with the 90 images mentioned above and again presented randomly. Their scores were used for measuring intrarater agreement. Thus, 108 images in total were analyzed. All images were compared during the analysis to a “reference” image chosen at random from a set of images from the manufacturer with the median quality grade in the kennel club database. In this way the VGA used can be described as a “relative VGA.”

Five VGA image criteria as follows were used. Criteria “A” and “C” were concerned with contrast resolution and low contrast resolution, respectively. Assessment of criterion “A” compared the demarcation between medullary and compact bone (mid diaphysis right femur) in the test image to that of the reference image. Criterion “C” compared the visualization of the acetabulum as it summates with the femoral head on the test image to that of the reference image. Criteria “B” and “E” were concerned with spatial resolution. Assessment of criterion “B” compared the sharpness of bone trabecula in the right femoral neck and greater trochanter area in the test image with that of the reference image. Assessment of criterion “E” compared the sharpness of the right femoral head on the test image with that of the reference image. Criterion “D” was concerned with image noise. Assessment of this criterion “D” compared the homogeneity of the soft tissues lateral to the mid diaphysis of the right femur with that of the reference image. These various image criteria were chosen to be relevant to the imaging task at hand, namely the evaluation of pelvis radiographs for hip dysplasia screening, and to correlate with those reported for similar imaging tasks in the literature (16). Scores were awarded on a 5 point scale, with scores of −2, −1, 0, +1, and +2 to indicate that a criterion is, respectively, much worse than, worse than, similar to, better than or much better than the same criterion on the reference image The mean of the 5 individual image criteria scores was determined for each image for each reader, and the mean of these reader scores was taken as the overall VGA score for each image. The score for each image is thus a mean of means. The minimum score an image could receive was −2, the maximum +2.

### 2.2. Viewing and Assessment

Three observers performed the assessment, a veterinary imaging resident, a veterinary radiologist and a human certified reporting radiographer at two different viewing locations (University of Copenhagen and Odense University). At both locations images were viewed on paired DICOM standard screens using DICOM display software (ViewDex V2.48) which has been used and described in observer performance studies in radiology (17, 18). This allowed the observer to view the test and the reference images side by side, to zoom, pan, alter window level and width for each image, and to enter the assessment for each parameter using a check box available on the side of the image. Responses are automatically logged in data files for the program. Images were presented in random order by the software; there was no opportunity to revisit images already scored. The observers could interrupt their session at any time and subsequently pick up where they left off. Each observer completed the task in 2 to 3 sessions.

### 2.3. Data Processing and Statistical Analysis

Data was extracted from image files using the PyDicom package (Version 1.2., available at https://pydicom.github.io/pydicom/stable/index.html) in Python (Version 3.7.2. Python Software Foundation, http://www.python.org). A Kruskal-Wallis rank sum test was used to test for associations between image quality grades from the kennel club database and the manufacturers. Differences in VGA test scores between the two quality groups were tested with a Wilcoxon rank sum test. Results of the VGA were explored using principal component analysis (PCA). Intraclass correlation coefficients for each observer were calculated to estimate repeatability of the VGA. Kendall's coefficient of concordance (Kendell's W) was used to the measure the degree of association between the assessments made by the three evaluators. All statistical tests and procedures, and the plot generation were performed using the statistical programming environment R (R: A Language and Environment for Statistical Computing, version 3.5.1, 2018, https://www.r-project.org/).

## 3. Results

### 3.1. Overview of the Dataset

A total of 40 different manufacturer names were identified in the dataset. Images from 9 manufacturers were represented by 20 or less images. These manufacturers and images were excluded from further consideration. Images where no quality grade was available in the kennel club database were also excluded from consideration. This process of elimination resulted in 15,859 of the original 16,360 images available for further analysis. Of these 15,859 images, 12,685 (80%) had image quality “Grade 1,” 2157 (14%) had image quality “Grade 2” and 607 (4%) had image quality “Grade 3.” The mean quality grades for these 31 manufacturers ranged from 1 to 2.13. There was a statistically significant association between kennel club quality grade and manufacturer (*p* < 0.001). The images were thus grouped by manufacturer and groups were then ranked according to the mean quality grade for the manufacturer. High and low quality groups were thus created as described in the methods and a VGA was performed.

### 3.2. Visual Grading Analysis

The range of the VGA scores for the images in the “low quality” group (*n* = 45) was −1.73 to +0.67, with a median value of −1.2. The corresponding values for images in the “high quality” group (*n* = 44) ranged from −1.52 to +0.53, with a median value −0.53. This difference was statistically significant (*p* < 0.001). The image numbers per group are not equal as one image had to be rejected from the assessment. It was an elbow joint image that was accidentally inserted into the wrong database group during initial upload to the kennel club PACS.

**Figure 1:**
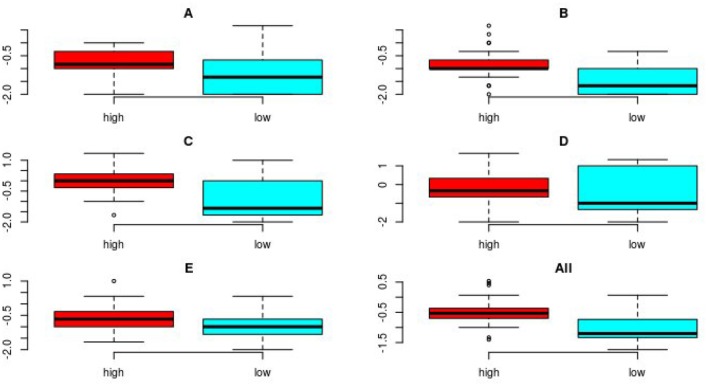
Boxplots showing the combined results of the VGA for the three readers for all images for each of the 5 image criteria, **(A–E)** and for all criteria combined, for both image groups (*n* = 44 and 45 for high quality/low quality, respectively). The boxes show the interquartile range of the data, the whiskers are set at 1.5 times the interquartile range or the maximum value and minimum values if these values are smaller or greater, respectively. The horizontal line shows the median of the data. Possible outliers are plotted individually outside the range of the whiskers. The median value for each image criterion evaluated and for all imaging criteria combined, was higher (indicating superior image quality) for the images in the high quality group compared to median value for images in the low quality group.

**Figure 2:**
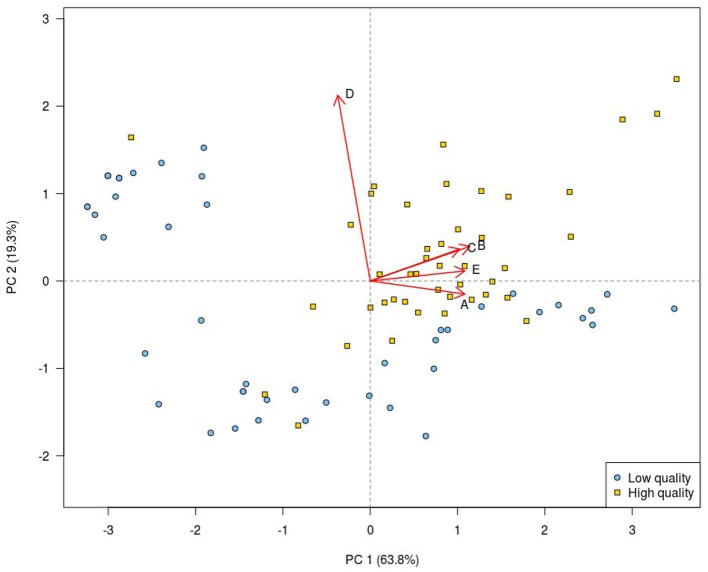
Principal component analysis biplot of the VGA results. Each plotting character represents a single image. Principal component analysis scores for the high quality images (yellow squares) are grouped in the upper right quadrant, while PCA scores for the low quality images (blue circles) mainly occupy the remaining quadrants. This indicates that the VGA appears successful in separating the two image groups. The first (PC 1) and the second (PC 2) principal components taken together describe 83.1% of the variation in the data. The loadings (red arrows) show the contribution of each image criterion and their degree of correlation. The biplot shows that image criteria A, B, C, and E were positively correlated, while there was poor correlation between the PCA scores for image criterion D and those of all other image criteria.

**Figure 3:**
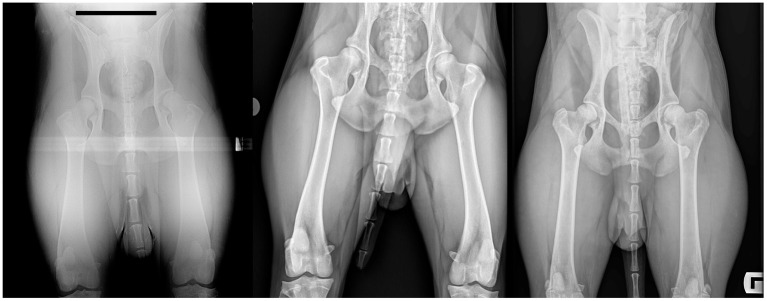
Test images selected according to VGA results, and the reference image. The relatively low quality image (left) has an overall VGA value of −1.7. It thus lies on the spectrum between being worse (−1) and much worse (−2) than the reference image. The relatively high quality image (right) has an overall VGA value of +0.5. It thus lies on the spectrum between being similar to and better than, the reference image. The reference image is shown in the center. The assessment criteria called on the observers to focus attention on the region of the mid-diaphysis of the right femur and on the right hip/femoral trochanter region. Images have been cropped and identifying data masked for this figure.

The scores for each image criterion are shown for each quality group in Figure 1. It can be seen that for all image criteria, images in the high quality group outperformed those in the low quality group. The PCA of the data shows that despite some overlap, there was a separation in images according to image quality group when the first and second principal components of the VGA data were plotted against each other. The loadings shown on the biplot (Figure 2) indicate that there was a positive correlation between the assessment of image criteria A, B, C, and E. These criteria are concerned with contrast resolution (criterion A), spatial resolution (criteria B and E), and low contrast resolution (criterion C). The assessment of criterion D (image noise) was not correlated to the other evaluation criteria. In the PCA, 83.1% of the variation in the data was explained by the first two principal components (PC1 and PC2). The reference image and examples of the test images are shown in Figure 3.

### 3.3. Intraclass Correlation and Kendall's W Coefficients

The ICC was determined for each readers assessment of duplicate images. The values of this test statistic for the three readers were 0.907, 0.921, and 0.948. Values above 0.9 are considered to indicate excellent agreement (19). The value of Kendall's W coefficient of concordance was 0.8 (zero indicates no agreement between raters; 1 indicates perfect agreement).

## 4. Discussion

This study set out determine if a suspected association between image quality and image detector manufacturer existed in a large cohort of images submitted for hip dysplasia screening to the Danish Kennel Club. The results of the initial analysis of the metadata from the images and their associated quality assessment indicate that one or more manufacturers are over-represented in one or more of the kennel club image quality grades. This demonstrates that there is an association between these quality grades and the system manufacturer associated with the image. The VGA was used to determine if an assessment based on image quality only, using carefully selected image criteria will also demonstrate differences according to manufacturer.

The evaluation criteria used in this study relate to detector and image processing performance and are particularly relevant to the evaluation of skeletal disease. With regard to the reference image, it is important to note that it should not be thought of as an ideal image. An optimal reference image from the point of view of the VGA will rank midway in quality with the test set images; some images in the test set will be found inferior to the reference, others superior. In this way if the reference image is ideal, the full range of test scores will be utilized in the assessment. The degree to which a reference image proves to be optimal only becomes clear as the study progresses, and only after an analysis of the results has been performed.

Choice of image criteria for evaluation is important. In this study image criteria A and C indicate contrast resolution, B and E are related to spatial resolution and criterion D relates to system noise. All were chosen with skeletal assessment in mind. Other relevant image criteria can be envisaged for assessment of other tasks, e.g., thoracic and abdominal imaging. In this study expert participation from academic radiographers and experience of veterinary imaging were combined to devise relevant image features for assessment by VGA. Well-chosen VGA criteria give rise to evaluations that are clinically relevant and allow an assessment process for the observers that is similar to their day to day clinical image evaluations.

The underlying technical influences on the outcome of the VGA have to be considered if system quality is found to be unsatisfactory. This consideration requires a record of the details of the image system and of the imaging parameters. This information is essential for remedial action. Details required will include focal spot size, degree of collimation, exposure tube current (mA) time (s), and kilovoltage (kVp), detector object distance, detector to focus distance, detector and anti-scatter grid specifications including fill factor, exposure index values, patient thickness, region examined and reconstruction algorithm used. If these parameters are known, a recommendation for image optimization can be made. Some of these data, specifically those relating to the x-ray generator and exposure factors, were not available to us in this study. Also the relative purchase costs of the equipment detector systems was not known. For this reason we cannot specify causes for the different image quality scores awarded. Of the list given here, many parameters will be constant within a practice, available in the system documentation or be self-evident from the image. It may be that only mA, s, kVp, patient thickness, exposure index values and collimation need to be recorded for each exposure by the radiographer for practical recommendations for improvement to be made.

The VGA results in this study show a significant difference in score between groups, indicating that veterinary imaging systems are not equal in terms of the image quality represented by their images in a large database. There are potential explanations for this. It may be that the hardware and software of one system are superior to those of another; it may be that systems are technically equal, but all do not run to manufacturer's specification either because qualified technical support is not available or not sought. There is some support for the latter view in that for most criteria shown in Figure 1, there is a greater variability in the VGA data for the low quality compared to the high quality group. The data therefore does not provide an ordered list of systems by quality to which manufacturers name can be fairly added. Such a list would require that comparisons were made between images of the same patient or object, a standardized technique and that all manufacturers confirmed that their systems were working and used according to their specification. The ranked list that such a study would produce could then be displayed along with equipment cost, or ranked lists could be grouped according to equipment cost. We feel, however, that given the number of images examined (almost 16,000 in the initial survey and 90 in the detailed VGA), bias that may be introduced by one patient type or other non-system variable is reduced. The high levels of agreement, with ICC values between 0.91 and 0.95, indicate excellent reliability for the VGA (20). Thus the data are a fair indicator of current status; an indicator that there is an association between image quality and manufacturer. It should also be noted that all the images included in the study were of diagnostic quality for the clinical indication at hand (hip dysplasia screening). However, other clinical scenarios can be envisaged where the shortfalls in spatial or contrast resolution or in image noise detected in the images examined could be limiting in diagnosis.

A further study that eliminated variance in radiographic technique e.g., standard radiographic subject and consistent radiographer and technique might refine the findings. Those findings might then, quite reasonably, be correlated with the costs of the installation as well as the manufacturer. It is also quite likely that one manufacturer may produce installations of differing complexity, cost, and image quality; this would have to be considered.

Veterinarians should be aware of inequalities as demonstrated in this study, in image quality between systems from different manufacturers. Such awareness and a knowledge of image quality analysis, particularly of relative VGA, would allow practitioners to make relevant quantitative image quality assessments as part of their purchasing, commissioning and quality control protocols. Expertise is available in the human radiography community and greater collaboration between veterinarians and this community would likely improve the general standard of quality control in veterinary imaging.

## Data Availability Statement

Anonymised datasets generated for this study are available on request to the corresponding author.

## Ethics Statement

The protocol was approved by the local Ethics and Administration Committee, Department of Veterinary Clinical Sciences, University of Copenhagen.

## Author Contributions

LM, HP, FM, and ES contributed to the conception and design of the study. HFP and FM organized the database, JJ, LM, and FM performed the VGA. HP, LM, JJ, and FM wrote the manuscript. FM performed the statistical analysis. ES and DN performed the initial image evaluations. All authors contributed to manuscript revision, read and approved the submitted version.

### Conflict of Interest

LM occupies an ANTECH Imaging Services funded European College of Veterinary Diagnostic Imaging residency at the University of Copenhagen. The funder was not involved in the study design, collection, analysis, interpretation of data, the writing of this article or the decision to submit it for publication. HFP was employed by the The Danish Kennel Club. The remaining authors declare that the research was conducted in the absence of any commercial or financial relationships that could be construed as a potential conflict of interest.
